# Experimental Evaluation of the Hypersensitivity Reactions of a New Glycopeptide Antibiotic Flavancin in Animal Models

**DOI:** 10.3390/ph16111569

**Published:** 2023-11-07

**Authors:** Michael I. Treshchalin, Vasilisa A. Polozkova, Elena I. Moiseenko, Andrey E. Shchekotikhin, Svetlana A. Dovzhenko, Mikhail B. Kobrin, Eleonora R. Pereverzeva

**Affiliations:** 1 Gause Institute of New Antibiotics, 11 B. Pirogovskaya Street, 119021 Moscow, Russia; vasilisa2006@gmail.com (V.A.P.); moiseenko.alena@gmail.com (E.I.M.); shchekotikhin@gause-inst.ru (A.E.S.); sad35@yandex.ru (S.A.D.); mbkobrin@gmail.com (M.B.K.); pereverzeva-ella@yandex.ru (E.R.P.); 2 Organic Chemistry Department, Mendeleyev University of Chemical Technology of Russia, 9 Miusskaya Square, 125047 Moscow, Russia

**Keywords:** glycopeptide antibiotics, flavancin, vancomycin, pseudo-allergic reactions, histamine release, anaphylactogenicity

## Abstract

Glycopeptide antibiotics are still in demand in clinical practice for treating infections caused by resistant gram-positive pathogens; however, their use is limited due to severe adverse reactions. Their predominant types of side effects are immunoglobulin E-mediated or nonmediated hypersensitivity reactions. Therefore, the development of new glycopeptide antibiotics with improved toxicity profiles remains an important objective in advancing modern antimicrobial agents. We investigated a new eremomycin aminoalkylamide flavancin, its anaphylactogenic properties, influence on histamine levels in blood plasma, pseudoallergic inflammatory reaction on concanavalin A and the change in the amount of flavancin in the blood plasma after administration. It has been shown that flavancin does not demonstrate anaphylactogenic properties. The injection of flavancin resulted in a level of histamine in the blood three times lower than that caused by vancomycin. The therapeutic dose of vancomycin led to a statistically significant increase in the concanavalin A response index compared to flavancin (54% versus 3.7%). Thus, flavancin does not cause a pseudo-allergic reaction. The rapid decrease in flavancin concentration in the blood and the low levels of histamine in the plasma lead us to assume that any pseudoallergic reactions resulting from flavancin application, if they do occur in clinical practice, will be significantly less compared to the use of vancomycin.

## 1. Introduction

Although the first representative of glycopeptides, vancomycin, was discovered in the 1950s, antibiotics of this class still remain in demand in clinical practice due to the continued increase in the number of infections caused by resistant gram-positive pathogens [1]. Glycopeptide antibiotics are commonly used for the treatment of diseases caused by these bacteria, but their use is limited due to severe adverse reactions [2]. The side effects of glycopeptide antibiotics include organ toxicity, such as nephrotoxicity, Ref. [3] as well as immunoglobulin E (IgE)-mediated (anaphylaxis) or nonmediated (red man syndrome) hypersensitivity reactions [4]. These two predominant types of side reactions share common clinical features and are characterized by an immediate onset, typically within 1 h after intravenous administration. In recent years, it has been established that vancomycin also exhibits a delayed toxic effect, which manifests as severe hypersensitivity, including conditions like chronic, acute generalized exanthematous pustulosis (AGEP), linear IgA bullous disease (LABD), and other bullous eruptions.

Non-IgE-mediated hypersensitivity to vancomycin, known as “Red Man Syndrome”, occurs in 4–47% of patients treated with vancomycin [4]. This reaction is typically associated with intravenous administration of doses exceeding 1000 mg and administrated at a rate of less than 60 min [5]. It is explained by vancomycin’s ability to activate mast cells directly, bypassing the action of IgE. The direct activation of mast cells by vancomycin was identified in rat models in the early 1990s [6,7]. More recently, the mechanism behind some IgE-independent reactions, previously referred to as pseudo-allergic or anaphylactoid reactions, has been further elucidated [8]. In vitro studies conducted by Azimi et al. demonstrated that vancomycin directly activates mast cell degranulation through the X2 receptor MRGPRX2 [9]. Activation of the MRGPRX2 receptor on mast cells leads to various proinflammatory mediators being released, including histamine [10,11].

In a number of randomized clinical trials, when measuring the concentration of histamine in plasma and vancomycin in the blood serum of patients and volunteers, it was found that a 1-h infusion is accompanied by a significantly higher peak plasma histamine concentration and greater total histamine release compared with a 2-h infusion [12]. There was no linear relationship between the concentration of vancomycin in the blood and the release of histamine. When vancomycin was administered over 2 h, along with a reduction in the amount of histamine released, a decrease in the frequency and severity of “Red Man Syndrome” was noted compared with a 1-h infusion. Thus, vancomycin-induced “Red Man Syndrome” was found to be mediated by histamine release and its severity correlates with the concentration and residence time of histamine in the blood plasma. A linear relationship between the concentration of vancomycin in the blood and the release of histamine has not been established. This is probably due to the fact that the relationships between the concentrations of vancomycin in blood serum and tissues, “vancomycin receptors” on basophils and mast cells, histamine concentrations, histamine receptors and the occurrence of “Red Man Syndrome” are complex and, apparently, vary greatly from person to person [13]. Although most hospital protocols require that vancomycin be administered for at least 60 min, there is still disagreement about the optimal dosage regimens for vancomycin [4,13]. Currently, attempts to optimize the use of vancomycin based on its pharmacokinetic parameters, in particular the use of the area under the total concentration–time curve divided by the minimum inhibitory concentration (AUC 24 /MIC) to determine the optimal dose, are ongoing [14,15,16]. Taking into account the pharmacokinetic/pharmacodynamic properties of vancomycin, it is possible not only to improve its antibacterial effectiveness but also to minimize the side effects.

In the course of our previous research focused on the development of new glycopeptide antibiotics with improved toxic properties and targeting antibiotic-resistant pathogens, we identified a promising candidate, eremomycin aminoalkylamide, called flavancin, for further in-depth preclinical studies. Flavacin showed advantages over the natural antibiotics vancomycin and eremomycin both in specific antibacterial activity and toxicological properties [17,18]. However, despite these positive findings, important aspects of flavancin, such as its potential to induce hypersensitivity reactions, remained unexplored. Therefore, the objective of this study was to investigate the ability of flavancin to induce the development of anaphylaxis in guinea pigs, the impact of flavancin on histamine levels in the blood plasma of mice and pseudoallergic inflammatory reaction on concanavalin A. In addition, in a trial on rats, plasma flavancin concentrations after intravenous administration were researched, which, in our opinion, could help in interpreting the results of the hypersensitivity reactions study.

## 2. Results

### 2.1. Anaphylactogenic Properties of Flavancin

The experimental design and results are presented in Table 1. Ten minutes after the injection of the resolving dose of cattle serum, all animals exhibited symptoms such as salivation and scratching. After 15–20 min, sneezing was observed. After 30–35 min, the majority of animals in the group experienced first tonic and then clonic convulsions. After 1 h, all animals assumed a sideways position and exhibited rapid breathing. Approximately one and a half hours after cattle serum administration, most animals developed abnormal Cheyne–Stokes respiration. After two hours, eight out of 10 pigs succumbed to the reaction. The remaining two pigs died the following day. In contrast, when flavancin was administered at any of the sensitizing doses studied, it did not provoke anaphylactic reactions in any of the animals. All the animals treated with flavancin remained alive. Thus, flavancin does not induce anaphylactic shock in guinea pigs that received the drug in one and three therapeutic doses (0.5 or 1.5 mg/kg).

Furthermore, flavancin does not induce active cutaneous anaphylaxis. The diameter of the blue spot observed in animals from both experimental groups did not show significant differences compared to the control group (Table 2).

Therefore, the finding demonstrates that flavancin does not possess anaphylactogenic properties and does not induce active skin anaphylaxis when administered at therapeutic dose and a dose exceeding the therapeutic dose by three times.

### 2.2. Histamine-Releasing Effect of Flavancin and Vancomycin

In our experiment, the concentration of histamine in the blood plasma of control mice was <0.4 µg/L. The use of vancomycin in MTD of 300 mg/kg led to the release of histamine in the blood plasma in an amount of 120.29 ± 3.12 µg/ L. After one minute, the level of histamine in the blood slightly increased and remained within these limits for the next 1.5 min (Table 3).

In contrast to vancomycin, when using flavancin at an equitoxic dose of 100 mg/kg, the level of histamine in the blood plasma after 30 s was 549.9 ± 92.6 µg/L. Within 1 min, it increased ~1.5 times, and then sharply decreased by ~2.5 times. At the same time, for this measurement period, the histamine concentration in animals in the group receiving flavancin was almost two times higher than in mice receiving vancomycin.

The administration of vancomycin at a therapeutic dose of 7.5 mg/kg also led to elevation of histamine levels in the blood plasma. Within 30 s after treatment, the histamine level peaked at 112.6 ± 27.8 µg/L. Subsequently, it rapidly decreased, reaching 13.4 ± 9.3 µg/L after 1.5 min (Table 4).

The administration of flavancin at an equivalent dose resulted in a significantly lower level of histamine in the blood after 30 s. Specifically, the histamine level was three times lower compared to vancomycin. Furthermore, after 1.5 min, the histamine level in the flavancin group did not show any significant difference from the control group.

### 2.3. Pseudoallergic Reaction

Table 5 shows that intravenous administration of vancomycin led to statistically significant elevations of Me with respect to Mc. The reaction index in these groups (RI) was 63% (MTD) and 54% (TD), respectively. In mice treated with flavancin, the values between Me and Mc were not statistically significant. The RI in these groups was only 3.7 (TD) and 7.3 (MTD) %, respectively.

Since the RI in the groups of mice treated with flavancin was less than 20%, it can be concluded that this drug did not induce a pseudoallergic reaction.

### 2.4. The Concentration of Flavancin in the Blood Plasma of Rats after Intravenous Administration

**Figure 1:**
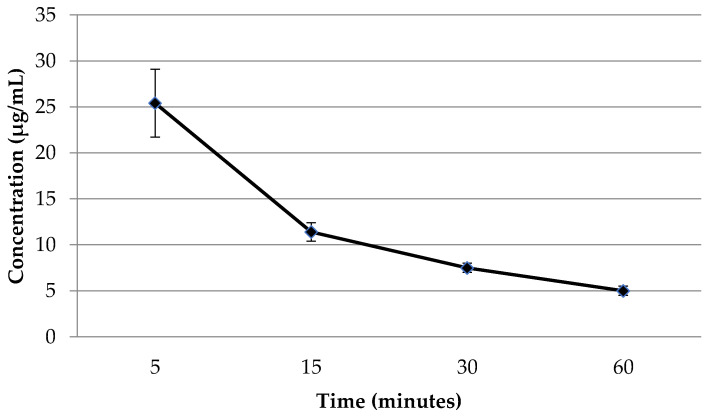
Plasma flavancin concentration (µg/mL) after intravenous administration in 5 therapeutic doses (5TD).

Finally, we determine the flavancin concentrations in rat plasma by using a high-performance liquid chromatography (HPLC). The results of the study, which examined the concentration of flavancin (µg/mL) in the blood plasma of rats after intravenous administration at a dose of 6.25 mg/kg (5 TD), are presented in Figure 1.

As can be seen from the data presented, the concentration levels of flavancin in the blood after administration quickly decrease within 1 h from 25 to 5 µg/mL.

## 3. Discussion

Our studies on flavancin have demonstrated that the drug, similar to vancomycin, triggers the release of histamine, resulting in an increase in its levels in the blood plasma within 30 s after administration. However, this release of histamine does not appear to be associated with IgE production in response to the antibiotic administration. This conclusion is supported by the fact that flavancin does not induce general anaphylactic reactions (anaphylactic shock) or active skin anaphylaxis, indicating the absence of anaphylactogenicity.

It is known that vancomycin rarely causes IgE-mediated reactions [19]. A recent systematic review by Minhas et al. on immune-mediated reactions to vancomycin found that approximately 10% of reported cases met the criteria for possible IgE-mediated mechanisms [20]. However, Hall et al., in a national study investigating the epidemiology of severe antibiotic-associated anaphylaxis in Australian hospitals, reported no cases of severe anaphylaxis associated with vancomycin [21]. In other studies, drug-dependent antibodies were not detected in blood samples collected after vancomycin treatment [22].

Reactions mediated by IgE antibodies, which are produced against drugs, are considered true allergies [23]. In these cases, the antibodies bind to the drug and IgE receptors on mast cells, leading to receptor cross-linking and intracellular signaling, resulting in the release of mediators such as histamine [9]. However, certain drugs, including vancomycin, morphine, curare, and others [24], can trigger mediator release by activating MRGPRX2 on mast cells.

Given that flavancin induces histamine release into the blood without displaying anaphylactogenic properties, one can expect the development of pseudo-allergic reactions. However, these reactions were not expressed since their intensity depends on the extent of histamine release. With both vancomycin and flavancin, plasma histamine concentrations depended on the dose administered, with toxic doses (MTDs) resulting in the release of significant amounts of histamine within 30 s. At the same time, under the influence of vancomycin, the amount of histamine released was significantly less compared to flavancin and remained at approximately the same level throughout the entire observation period. On the contrary, within 3 min after the administration of flavancin, the histamine level first increased sharply and then fell sharply, still remaining approximately two times higher compared to the vancomycin group.

Reducing the dose of drugs by approximately two orders of magnitude had a significant effect on the level of histamine and the dynamics of changes in its concentration in the blood. When using vancomycin in one TD after 30 s, the amount of histamine in the blood plasma was approximately the same as when it was administered at a dose equivalent to the MTD (120.3 versus 112.6 μg/ mL). However, in the next 2.5 min, it dropped to 13 μg/mL. The increase in histamine concentration under the influence of one TD flavancin was an order of magnitude less than when it was used in MTD (549 versus 33), and after 2.5 min it did not differ from the control. This is probably due to a rapid decrease in the concentration of flavancin in the blood.

Our experiment showed that within an hour, its amount decreases from 25 to 5 μg/mL, whereas, according to the literature, 1 h after a 60-min intravenous infusion of vancomycin at a therapeutic dose, its concentration in the blood decreases only two times (51.1 ± 9.2 versus 24.9 ± 2.9) [12].

It is precisely this dynamics of elimination of flavancin from the blood that can explain the absence of a pseudoallergic reaction to Con A in mice when it was used in both doses studied.

Based on the opinion of McNeil BD et al. [10] that secretagogues (substances that, by activating receptors, cause the release of another substance, in this case vancomycin or flavancin) activate mast cells only in high concentrations, it can be assumed that either the concentration of the drug is too low, or the time during which the concentration reaches levels that can effectively activate MrgprB2 receptors in mice, too small, and therefore the drug does not have time to trigger the symptoms of an anaphylactoid reaction.

In summary, we have shown that flavancin affects histamine release in a dose-dependent manner: the higher the dose, the more histamine is released. The duration of the presence of this inflammatory mediator in the blood is extremely short, and the lower the dose, the shorter the time that histamine remains in the blood. Apparently, due to the short duration of flavancin’s presence in the blood and its low concentration, the activation of specific receptors on the surface of mast cells and basophils does not have time to start, leading to the release of histamine, which induces an anaphylactoid reaction. The properties of flavancin, studied in experiments on animal models, showed its advantages over vancomycin. Although we understand the limited possibility of directly transferring the obtained experimental results to humans, in particular due to the difference in the properties of the G-protein coupled receptor MrgprX2 of the human and its orthologue MrgprB2 of mice, we hope that the new glycopeptide will not induce the development of pseudoallergic reactions in humans.

## 4. Materials and Methods

The studies of the anaphylactogenic properties, histamine-releasing effect, pseudoallergic reaction to Con A and flavancin concentration in the blood were conducted using adult animals received from the laboratory animal nursery of Scientific Center for Biomedical Technologies of the Federal Biomedical Agency (Moscow, Russia). The experiments were conducted in compliance with the European Convention for the Protection of Vertebrate Animals [25] and the Russian Federation standard «The Principles of Good Laboratory Practice» [26]. The ethical aspects of animal experimentation were reviewed and approved by the local ethics committee of the Gause Institute of New Antibiotics, with protocol number 03/2021 dated 12 March 2021.

Flavancin-N-(2((2-fluorobenzyl)amino)ethyl)amide of eremomycin hydrochloride, was synthesized using the method described earlier [17]. In chemical structure, it is close to vancomycin.

### 4.1. Anaphylactogenic Properties of Flavancin

In order to determine the anaphylactogenic properties of flavancin, we selected two technically simple and easily reproducible tests: general anaphylaxis reaction and active skin anaphylaxis. They have undergone long-term testing in the experimental assessment of the sensitizing properties of drugs. Their results correlate satisfactorily with the results of clinical studies in humans, which is why these tests are recommended as the main ones in the guidelines for preclinical studies.

#### 4.1.1. Generalized Anaphylaxis (Anaphylactic Shock)

The study was conducted on guinea pigs, because this species is known to easily develop systemic anaphylaxis [27]. Adult guinea pigs weighing 300–350 g were randomized into three groups (n = 10). The study utilized sensitizing doses of 0.5 mg/kg (1TD) and 1.5 mg/kg (3TD), as well as resolving dose of 3.0 mg/kg (6TD). These doses were determined based on the effectiveness in mice (ED_100_), with adjustments made using the coefficient of the animals’ body surface [28]. Saline was used as the control in the experiment. As for the reference agent, cattle serum was employed, with a dose of 0.1 mL for sensitizing doses and 0.5 mL for the resolving doses. The sensitizing doses were following a scheme: the first injection was given subcutaneously, while the subsequent two injections were administrated intramuscularly, with a 48 h interval between each injection. The resolving dose was administered intracardially 21 days after the last day of sensitization.

#### 4.1.2. Active Skin Anaphylaxis

In the study of active skin anaphylaxis, the sensitizing doses of flavancin were administered following the same scheme as described previously. The resolving dose, which consisted of a 0.1% solution of the dosage form of the drug in a 5% glucose solution with a volume of 0.05 mL per guinea pig, was administered intradermally 21 days after the last day of sensitization into a previously excised area of the flank skin. After 30 min, both the experimental and control groups of animals were intracardially injected with 0.5 mL of 1% Evans blue solution. Thirty minutes later, the animals were euthanized using ether anesthesia. The skin where the drug was intradermally administrated was then separated, and the diameter of the blue spot was measured and recorded. The collected quantitative data were statistically analyzed using a significance level of *p* < 0.05.

### 4.2. Determination of Histamine in Blood Plasma

The dependence of the intensity of histamine release and drug-associated systemic pseudoallergic or anaphylactoid reactions on the dose of the drug was shown in both experimental and clinical studies [10]. For determining the dependence of histamine level in the blood plasma on the concentration of the administered drug, two dose levels of flavancin and vancomycin were studied: toxic and therapeutic. The toxic dose was equivalent to the MTD (LD_10_) for mice (300 mg/kg for vancomycin and 100 mg/kg for flavancin). Therapeutic doses (ED_100_) for these drugs were determined by studying the effectiveness in a model of staphylococcal sepsis in mice and were 7.5 (vancomycin) and 2.5 mg/kg (flavancin) [17]. The amount of histamine in the blood plasma of mice was detected 0.5, 1.5 and 3 min after a single intravenous administration of drugs. Elevation in plasma histamine levels during anaphylaxis is very transient. It can be measured within a few minutes of release because the half-life of histamine in a biological system before it is converted to n-methylhistamine is about four minutes before conversion to n-methyl histamine [29]. Normally, there is a minimal amount of histamine circulating in the body of human or animals.

Female CBA mice weighing 18–20 g were divided into groups of 10 animals each. Flavancin was dissolved in 5% glucose and administrated via the tail vein to the mice at a dose equivalent to one TD (2.5 mg/kg) or MTD (100 mg/kg). Vancomycin (Vancomycin-Teva, Petach-Tikva, Israel) was used as the reference drug and administrated at an equitherapeutic dose of 7.5 mg/kg or equitoxic dose of 300 mg/kg. Untreated animals were used as the control group.

The animals were euthanized after 0.5, 1 and 1.5 min in an experiment using equiterapeutic doses and 0.5, 1.5 and 3 min for an experiment with the use of equitoxic doses, and the blood samples were collected after the drug administration. Next, 500 µL of whole blood, which had been drawn into 10 µL of heparin (30–50 IU) was centrifuged for 20 min at 2000 rpm. To perform the histamine analyses, 20 µL of plasma was mixed with dilutions of saline at ratios of 1:10 and 1:20. The amount of histamine was determined using an automatic biochemical and enzyme immunoassay analyzer (ChemWell, Awareness Technology, Inc., Palm City, FL, USA). The histamine detection was performed using the Histamine-ELISA reagent kit (Tecan Group Company, Männedorf, Switzerland).

### 4.3. Evaluation of Pseudoallergic Reaction

We compared the ability of flavancin and vancomycin to cause a pseudoallergic reaction in a test with Con A. The test is based on the induction of an inflammation in response to the direct interaction of Con A with the receptors of mast cells and basophils. Its administration causes nonimmune activation of histamine release and inflammatory edema at the injection site.

The experiment was carried out in accordance with «Guidelines on the assessment of allergic properties of drugs» [30]. Healthy female CBA mice weighing 20–24 g were divided into five groups, in cages of 10. Solutions of flavancin and vancomycin in 5% glucose were administered intravenously in MTD and TD doses. 10 mL/kg of 5% glucose solution was administered to the animals of control group. One hour post-injection, 0.5% Con A in isotonic sodium chloride solution (0.01 mL/10 g body mass) was injected into the pad of the hind legs and the same volume of isotonic solution of sodium chloride was injected into the contralateral extremity of mice in the experimental and control groups. One hour later, the mice were euthanized. The hind legs were amputated at the hock joint and weighed. Relative increase Me over Mc in each group was calculated using the formula (Me − Mc)/Mc × 100%, where Me is the mass of the foot injected with Con A and Mc is the mass of the foot injected with saline solution. The RI of the group of animals receiving the drug, exceeding 20%, is considered significant and indicates an inflammatory reaction to concanavalin A.

### 4.4. Determination of Flavancin Concentrations in Rat Plasma by a High-Performance Liquid Chromatography

The study was carried out on Wistar male rats weighing from 180 to 220 g. Experimental animals were divided into groups of six at each time point. Flavancin was administered intravenously into the tail vein at a single dose of 6.25 mg/kg (5 TD). Control rats were injected with glucose solution in the same volume. Blood was collected 5, 15, 30 min and 1 h after injection into heparinized plastic tubes. Blood samples were centrifuged for 20 min at 1500 rpm. The supernatant was poured into tubes and stored at −20 °C until chromatographic analysis. Quantitative determination of flavancin in blood samples was carried out by high-performance liquid chromatography method (HPLC) with fluorimetric detection.

The method is based on the chromatography of specially prepared samples using a high-performance liquid chromatograph with a fluorescence detector and a reversed-phase column.

#### 4.4.1. Chromatography Conditions for Flavancin

Gradient separation was performed at 40 °C on a column Jupiter C18 (250 mm × 4.6 mm, particle size 5 microns; Security Guard Cartridge, Phenomenex, Torrance, CA, USA). The mobile phase A consisted of a 100 mM solution of potassium dihydrogen phosphate, adjusted to pH 3.15 with phosphoric acid (buffer). The mobile phase B consisted of a buffer and acetonitrile in a volume ratio of 1:1, flowing at a rate of 1.0 mL/min. The following mobile phase gradient was used: the proportion of MP B was increased from 0% to 18% over 5.0 min, then increased to 21% from 5.0 to 8.5 min, then increased to 32% from 8.5 to 12.0 min, further increased to 100% from 12.0 to 13.0 min, then isocratic elution 100% till to 17 min, after which it was returned to the initial composition and equilibrated until 12.0 min. The sample volume was 20 µL. Vancomycin was used as an internal standard (IS). Detection was carried out using a fluorescence detector (Waters 2475, Waters Associates, Framingham, MA, USA) (excitation—240 nm, emission—330 nm). The retention times of IS and flavancin were 13.5 and 15 min, respectively. The relative standard deviations (n = 5) for the concentrations of flavancin of 51.9, 10.4, 5.2 and 0.8 µg/mL were 2.6, 3.3, 5.9, and 8.5%, respectively. The calibration graphs were linear (r^2^ ≥ 0.99) in the range of flavancin concentrations from 0.8 to 52.0 µg/mL. The lower limit of the quantitative determination of flavancin was 0.8 µg/mL. The limit of detection was 0.3 µg/mL. Recovery was 31.5% for flavancin and 78.5% for IS.

#### 4.4.2. Sample Preparation

Two hundred and fifty µL of rat plasma was placed in 1.5 mL Eppendorf plastic tubes; 100 µL of vancomycin physiological saline solution (250 µg/mL) was added, shaken for 1 min, then 500 µL acetonitrile was added, shaken for 2 min, and centrifuged for 5 min at 13,000 rpm at 30 °C. Eight hundred µL of supernatant was replaced in 1.5 mL Eppendorf plastic tubes. The tubes were placed in a vacuum centrifuge (SC210A, Savant, Hyannis, MA, USA) and evaporated to a volume of 150–200 µL at room temperature. The resulting solutions were placed in an ultrasonic bath for 10 s at 40 °C and then centrifuged for 5 min at 13,000 rpm at 30 °C. The supernatant was analyzed by HPLC.

#### 4.4.3. Preparation of Standard Samples

Solutions of flavancin for constructing the calibration dependence were obtained by sequential redilution of the initial solution of the analyte in blank plasma. The resulting solutions were processed as described in sample preparation procedure.

Statistical analysis of the obtained results was carried out using Student’s *t*-test. Mean values and standard deviations were calculated, and a significance level of *p* ≤ 0.05 was considered to determine the difference between the groups. The StatPlus 2006 v. 3.8.0.0. AnalystSoft computer program was used for the statistical processing of the data.

## 5. Conclusions

New eremomycin aminoalkylamide flavancin does not possess anaphylactogenic properties. It does not cause general anaphylaxis reaction (anaphylactic shock) or active skin anaphylaxis, but induces the release of histamine, which is probably due to the direct interaction of the compound with mast cell receptors; thus, it is IgE-independent. Apparently, flavancin in both doses studied did not induce a pseudoallergic reaction on Con A in mice due to the short duration of flavancin’s presence in the blood and its low concentration. Overall, the data obtained suggest that any anaphylactoid reactions resulting from flavancin application, if they do occur in clinical practice, will be significantly less compared to the use of vancomycin.

## Figures and Tables

**Table 1:** General anaphylaxis reaction to flavancin (anaphylactic shock).

Sensitizing Doses, mg/kg			Resolving Doses, mg/kg	Effect
Subcutaneous	Intramuscularly	Intramuscularly	Intracardially	
flavancin	flavancin	flavancin	flavancin	no
0.5	0.5	0.5	3.0	
flavancin	flavancin	flavancin	flavancin	no
1.5	1.5	1.5	3.0	
saline solution	saline solution	saline solution	flavancin	no
1.0 mL	1.0 mL	1.0 mL	3.0	
cattle serum	cattle serum	cattle serum	cattle serum	Severe anaphylactic reaction. After two hours, 8 pigs out of 10 died. A day later, the remaining two pigs died.
0.1 mL	0.1 mL	0.1 mL	0.5 mL	

**Table 2:** Active cutaneous anaphylaxis to flavancin.

Sensitizing Doses, mg/kg			Resolving Doses	Blue Spot Diameter, cm
Subcutaneous	Intramuscularly	Intramuscularly	Intracardially	
flavancin	flavancin	flavancin	flavancin	2.3 ± 0.6
0.5	0.5	0.5	0.05 mL per pig	
flavancin	flavancin	flavancin	flavancin	2.4 ± 0.5
1.5	1.5	1.5	0.05 mL per pig	
saline solution	saline solution	saline solution	flavancin	2.5 ± 0.5
1.0 mL	1.0 mL	1.0 mL	0.05 mL per pig	

**Table 3:** Plasma histamine concentrations after flavancin and vancomycin administration in equitoxic doses (~MTD).

Agent	Dose, mg/kg	Time, Minutes	Histamine Level, µg/L
Vancomycin	300	0.5	120.3 ± 3.1
		1.5	145.0 ± 10.1
		3	170.6 ± 20.9
Flavancin	100	0.5	549.9 ± 92.6
		1.5	796.2 ± 56.3
		3	297.7 ± 54.9
Control			<0.4

**Table 4:** Plasma histamine concentrations after flavancin and vancomycin administration in equitherapeutic doses (1TD).

Agent	Dose, mg/kg	Time, Minutes	Histamine Level, µg/L
Vancomycin	7.5	0.5	112.6 ± 27.8
		1	85.2 ± 14.7
		1.5	13.4 ± 9.3
Flavancin	2.5	0.5	33.3 ± 7.0
		1	16.8 ± 7.7
		1.5	<1.1
Control			<1.1

**Table 5:** Reaction index (RI) to concanavalin A in mice treated with vancomycin and flavancin.

Drug	The Mass of the Hind Leg Foot (mg)		Relative Increase Me Over Mc, %	RI, %
	Con A Me Mean ± SD	Saline Mc Mean ± SD		
Vancomycin, MTD	149.4 ± 3.0	131.8 ± 3.6	13.4	63
Vancomycin, TD	158.1 ± 3.8	140.4 ± 2.3	12.6	54
Flavancin, MTD	151.5 ± 3.0	139.3 ± 2.9	8.8	7.3
Flavancin, TD	154.3 ± 2.1	142.1 ± 2.0	8.5	3.7
Control	148.2 ± 2.4	137.0 ± 2.3	8.2	–

Notes: Mc—the mass of the paw, into the pad of which saline solution was injected. Me—the mass of the paw, into the pad of which Con A was injected.

## Data Availability

Data is contained within the article.
